# Interfacial Chemistry Investigation of Initial Fouling Conditions in Isocyanate Production: The Antifouling Performance of AISI 316L Stainless Steel

**DOI:** 10.1021/acsomega.1c02711

**Published:** 2021-10-01

**Authors:** Clayton Bevas, Marie-Laure Abel, Ivo Jacobs, Karin van Oudgaarden, John F. Watts

**Affiliations:** † Department of Mechanical Engineering Sciences, University of Surrey, Guildford, Surrey GU2 7XH, U.K.; ‡ Global Excellence Team, Huntsman Polyurethanes, 3197 KG Rotterdam, The Netherlands

## Abstract

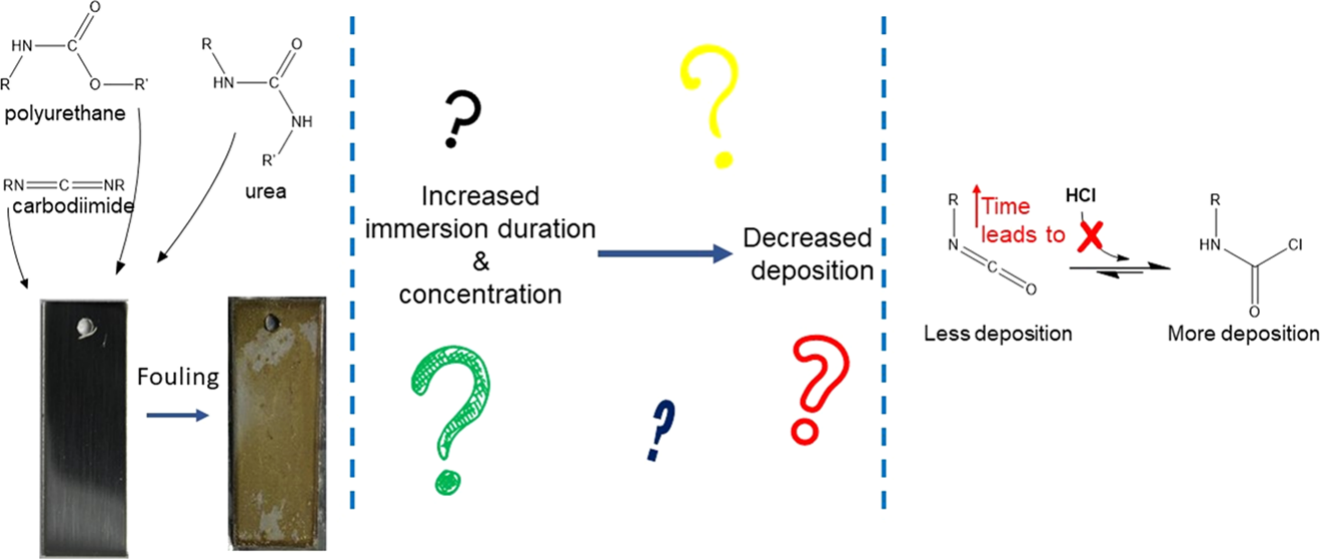

The fouling of AISI 316L stainless steel during themanufacture of polymeric methylene diphenyl diisocyanate (pMDI) has been investigated. Studies have been carried out using a laboratory-based rig that simulates the process chemistry of the production plant. A variety of solution concentrations and treatment times have been employed to represent different stages in the production process. Following exposure, steel coupons have been removed and studied by X-ray photoelectron spectroscopy (XPS) and time-of-flight secondary ion mass spectrometry (ToF-SIMS). The thickness of the fouling layer, determined by XPS, is found to vary inversely with exposure time and solution concentration. This is a result of the solubility of the different pMDI derivatives that have been formed at different stages, and a reaction scheme is developed to explain these inverse relationships. ToF-SIMS indicates the formation of metal chlorides as a result of the initial treatment of the steel in the reaction vessel with hydrogen chloride. Fragment ions characteristic of reacted and unreacted pMDI (at *m*/*z* = 106 and 132 au, respectively) were used as an indicator of the degree of reacted isocyanate groups within the fouling layer and show a decrease with increasing exposure time, as a result of the formation of intermediates such as amines, ureas, carbodiimides, and uretonimines. The ToF-SIMS data was also processed by principal component analysis (PCA). This generally reinforced the conclusions reached by XPS and ToF-SIMS but, in addition, gave confidence in the repeatability of the analyses with the repeat data (of four analyses) clustering very tightly in the PCA score plots.

## Introduction

Polyurethanes are a versatile group of polymeric materials with applications as elastomers, coatings, adhesives, and foams.^1,2^ Polyurethanes are produced through a polyaddition reaction between polyisocyanates and polyols. Methylene diphenyl diisocyanate (MDI) is one of the most common polyisocyanates used in the production of polyurethanes.

**Figure 1:**
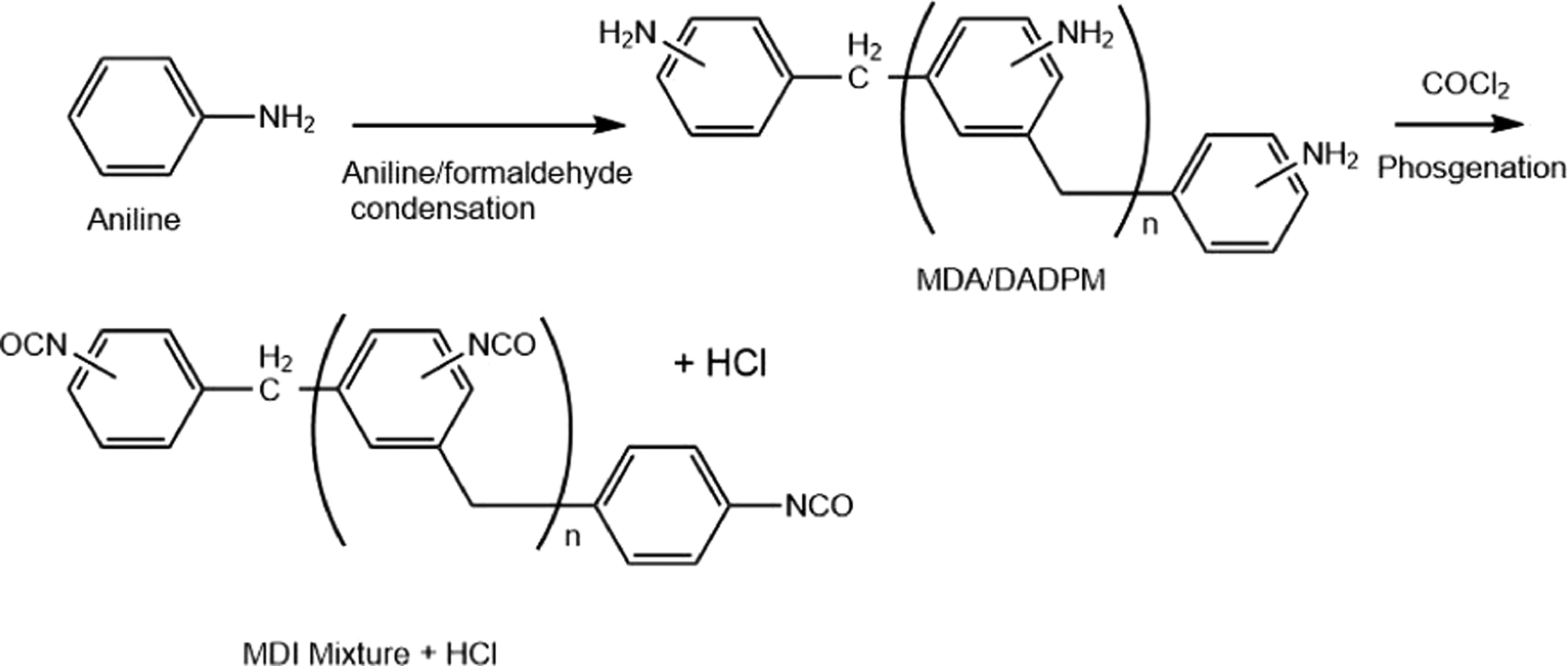
Reaction scheme detailing aniline polycondensation with formaldehyde and subsequent phosgenation in MDI production.

The MDI production process is described in detail elsewhere;^2,3^ however, the main steps involve the polycondensation of aniline with formaldehyde and subsequent phosgenation. This polymerization reaction is detailed in Figure 1. Additional isocyanate reactions relevant to this work are detailed in the Supporting Information. After phosgenation, crude MDI is obtained through a series of heat treatment steps used to remove excess reactants and solvents. Pure MDI and polymeric MDI (pMDI) are obtained from crude MDI through a distillation step. The heat exchangers used in the heat treatment and distillation steps are commonly made from 316L or Duplex stainless steels, chosen for their corrosion resistance properties. Fouling and degradation of the metal surface can still occur, however, reducing the efficiency of the heat exchanger, requiring periodic cleaning and the disposal of contaminated water and other residues to restore the metal surface.

AISI 316L stainless steel is a common austenitic stainless steel with increased resistance to crevice corrosion and improved weldability as a result of the addition of molybdenum and reduced carbon concentration, respectively. The corrosion resistance of 316L, like all stainless steels, comes from the stability of its chromium-rich passive film, which repassivates upon light damage, restoring its corrosion resistance.^4,5^ 316L finds use in a variety of sectors such as desalination, chemical production, pharmaceutical, portable water treatment, and other high-temperature applications.^6−8^

The interface chemistry of isocyanates and metal surfaces has been investigated by numerous research groups previously. In 1991, Chehimi and Watts investigated the interaction between moisture-cured polyurethanes and steel surfaces.^9^ Their investigation identified new chemical species formed at the interface, most likely involving iron oxide from the passive film and oxygen- and nitrogen-containing species from the polymer. Although not confirmed with certainty, results suggested that iron carbamate species were formed and migrated to the interface zone. Dillingham and Moriarty also proposed the formation of iron carbamate bonds during the adhesion process between isocyanates and steel in an infrared reflection spectroscopy study.^10^ Using infrared spectroscopic techniques, Nies et al. studied the adhesive interactions of MDI on Au, Al, and Cu surfaces.^11^ Chemisorption-type mechanisms were identified at the interfacial region of Al and Cu, while Au presented with physisorption-type bonding at the interface.

The thermodynamics of pMDI adsorption on aluminum and iron surfaces was also studied by Shimizu.^12^ For both aluminum and iron substrates, Langmuir isotherm adsorption behavior was observed over most adsorbent concentrations. However, at concentrations below 1 g L^–1^, adsorption did not follow the expected Langmuir isotherm. It was suggested that shorter pMDI molecules initially adhere to more favorable sites on the substrate surface at low concentrations. Once adsorbate concentrations increase, larger molecules begin to displace smaller ones and the system starts to show the characteristic Langmuir adsorption isotherm.

Tardio et al. have investigated interactions between MDI and 316L stainless steel with a focus on the identification of interfacial interactions. When pre-exposed to water, steel shows a greater affinity to MDI. It was concluded that a monolayer of MDI forms on the surface of the steel, bonding through a strong covalent bond between the isocyanate group of the MDI and the hydroxide group on the steel surface.^13^ Cycloaddition reactions between Fe=O and −N=CO were suggested based on time-of-flight secondary ion mass spectrometry (ToF-SIMS) data. Both the nucleophilic attack and the cycloaddition reaction are in keeping with the known chemistry of isocyanates.^3^

The work carried out by Bañuls-Ciscar et al. built upon the above work by Tardio et al. through an interfacial study of pMDI and an Fe–Cr alloy.^14^ Nitride-link bonds between iron and chromium in the metal and nitrogen from the pMDI were identified in their X-ray photoelectron spectroscopy (XPS) analysis. In addition to this, multiple interface-specific fragments were identified in non-negative matrix factorization analysis of a ToF-SIMS depth profile. These fragments were indicative of the same interfacial bonding structures identified by Tardio et al.^13^

Additional information regarding MDI chemistry relevant to the polymerization of MDI can be found elsewhere,^2^ and a brief review can be found in the Supporting Information.

This work aims to assess the initial stages of fouling in MDI production using a laboratory-scale system that replicates fouling conditions found in large-scale MDI production plants. As fouling in the production plant occurs over many months, testing carried out in the laboratory will be accelerated to mimic the extended operation time. XPS and ToF-SIMS are both surface-sensitive characterization techniques, which are used extensively in the work described in this paper. Their analysis depths are approximately 5 and 3 nm, respectively, depending on the sample type being analyzed and experimental conditions. This makes these techniques well suited to the study of interface chemistry. The quantitative properties of XPS enable the estimation of overlayer thicknesses and surface composition. ToF-SIMS spectra contain detailed information regarding the structure of organic species, and the high sensitivity of the technique readily enables the identification of organic and inorganic trace species. The interface chemistry between 316L stainless steel and the organic system will be examined and a mechanism for initial fouling will be proposed.

## Results and Discussion

### Visual Comparison

**Figure 2:**
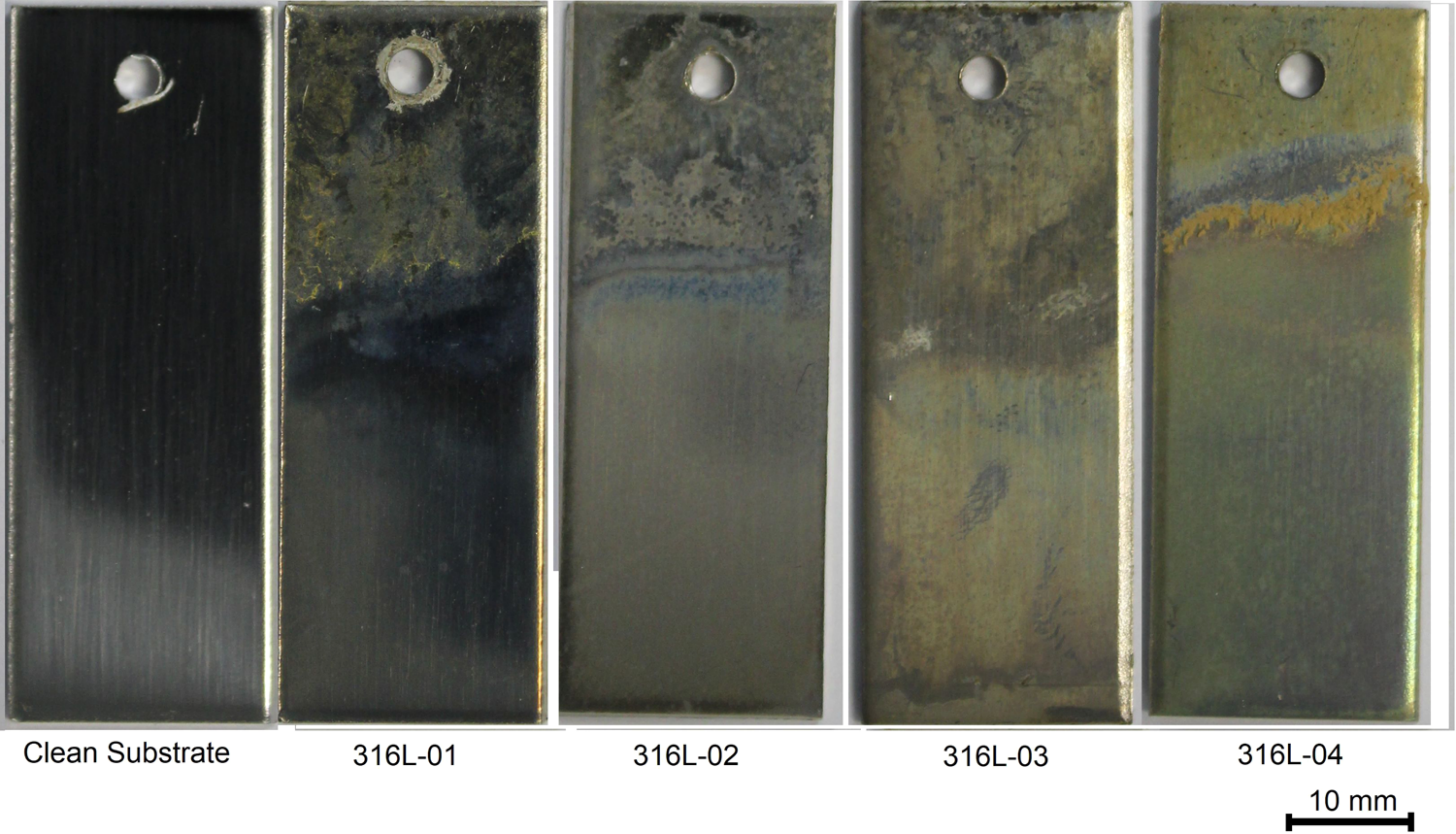
Appearance of all coated 316L samples and the clean 316L substrate prior to coating.

Images of all 316L samples are provided in Figure 2, and the sample size is 20 × 50 mm^2^. Pale yellow deposits are observed across the headspace region of all samples. The boundary between the headspace zone and the immersed region (referred to as the wash zone) is clearly seen in all of the exposed samples in Figure 2. These deposits are darkest on 316L-04, which also shows a highly fouled region across the wash region with solid deposits adhered to the surface. The coverage of these solid deposits appeared more extensive prior to rinsing with monochlorobenzene (MCB) and is assumed to be pMDI-based material adhered to the surface. The submerged regions have a pale yellow coverage across them, with the shortest immersion duration samples showing the darkest region.

A deeper color on the submerged region can correspond to an increase in the surface-bound organic material. This therefore suggests a trend of decreased surface coverage with increased immersion duration. This is counterintuitive as one would expect deposition to increase with increased immersion duration. In addition to this, the submerged region of 316L-04 is one of the darkest submerged regions; however, this was immersed in the lower concentration solution of pMDI, which is also a counterintuitive observation. To investigate these initial observations further, the XPS and ToF-SIMS analyses of these samples were conducted focusing on the submerged regions.

### XPS Analysis

**Figure 3:**
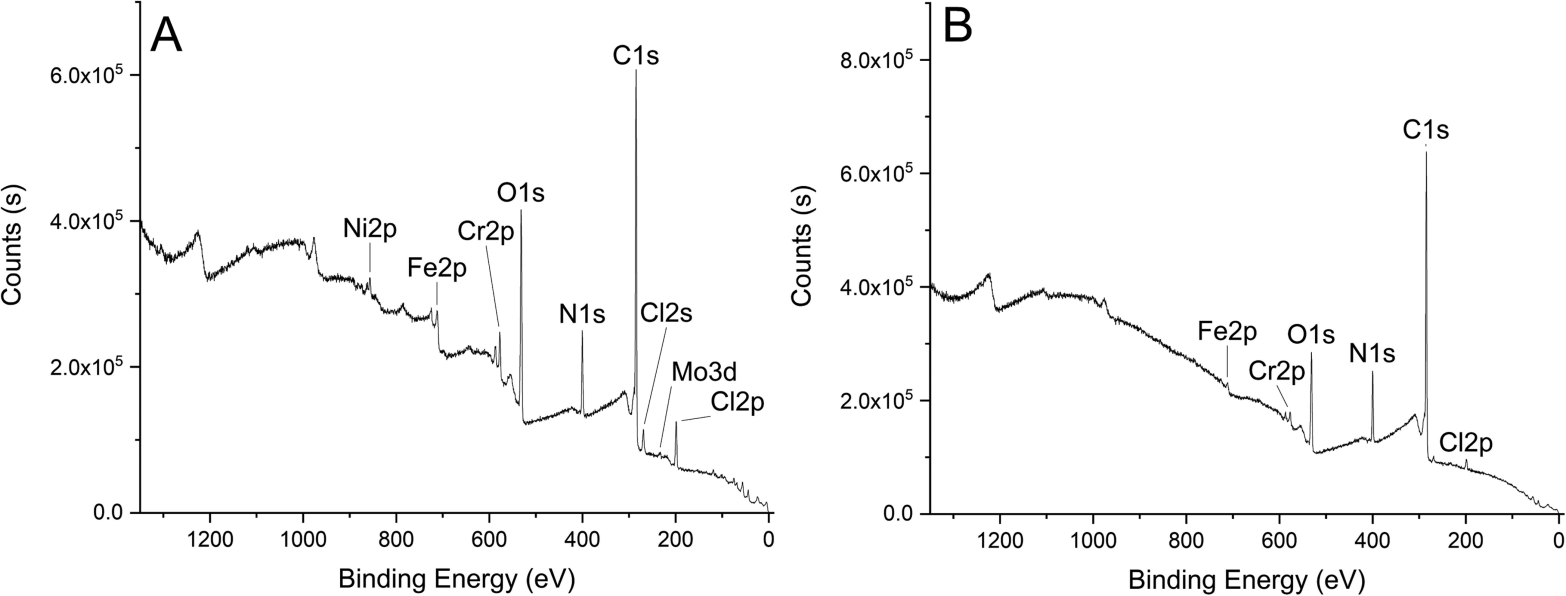
XPS survey spectra of (A) 316L-01 and (B) 316L-03.

**Figure 4:**
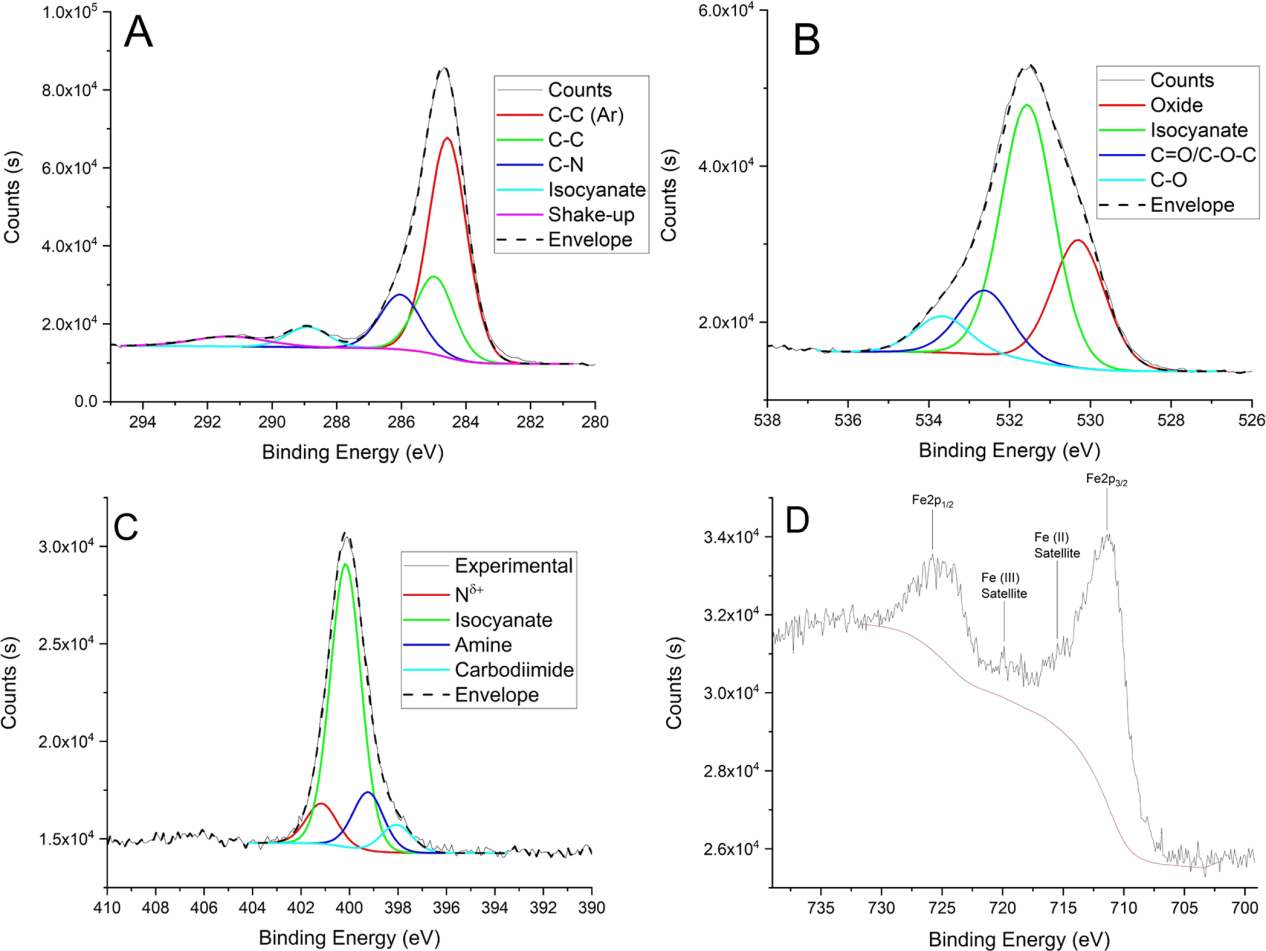
(A) C 1s, (B) O 1s, (C) N 1s, and (D) Fe 2p XPS spectra of 316L-01.

All survey spectra show surfaces indicative of pMDI (Figure 3). Carbon, nitrogen, oxygen, and chlorine are present in all samples. Iron, chromium, and molybdenum are present in 316L-01/02/03 and nickel in 316L-01/02. Rising backgrounds are observed to the higher binding energy side of the Cr 2p and Ni 2p peaks in 316L-01, indicating that these photoelectrons are inelastically scattered and these metals are primarily located underneath the organic overlayer. Similar trends are observed in 316L-03, where a rising background is observed following Fe 2p and Cr 2p peaks. Iron and chromium are mainly present in the (III) oxidation state. Satellites indicative of Fe(III) and Fe(II) are both present in the Fe 2p spectrum (Figure 4). The survey spectrum of 316L-04 is indicative of a thick (≫10 nm) pMDI overlayer. Carbon, oxygen, and nitrogen concentrations are consistent with pMDI, and no evidence of any metal species is present.

Peak fitting of high-resolution spectra was carried out using the Avantage v5.890 software package. Singlet peaks were added to C 1s, O 1s, and N 1s spectra based on the components present in previous studies of pMDI and related materials.^13−15^ Component binding energies were maintained from previous studies ±0.1 eV to enable the best fit. The full width at half-maximum (FWHM) value was kept constant for C 1s (1.4 and 2.3 eV for C–C/H and shake-up satellite and 1.5 eV for all other components), O 1s (1.6 eV), and N 1s (1.5 eV). Quantified surface concentrations for all samples can be found in Table 1.

**Table 1:** Quantified Surface Concentrations for All Samples

	surface concentration (atom %)							
sample	C	O	N	Cl	Fe	Cr	Ni	Mo
316L-01	64.6	18.1	9.2	3.2	2	1.7	1.0	0.1
316L-02	62.1	21	9.0	3.8	0.9	2.5	0.5	0.2
316L-03	74.5	12.4	10.7	1.0	0.6	0.7		0.2
316L-04	82.2	5.4	11.7	0.7				

The high-resolution C 1s, O 1s, and N 1s spectra for all samples are indicative of pMDI, with the isocyanate components clearly visible at 288.9, 531.4, and 400.2 eV in the C 1s, O 1s, and N 1s spectra, respectively (Figure 4). The C 1s spectrum of 316L-01 presents a C–N component larger than the NCO component. This is expected as solid pMDI deposits contain a mix of functional groups including isocyanates and amines. The C–N component is indicative of both species; however, the isocyanate component is only indicative of the former and therefore the C–N component is expected to be larger.

The NCO, C=O, and C–O components in the O 1s spectrum are indicative of pMDI. The additional oxide component is from the metal oxide passive film on the substrate and agrees with the assessment that the metal species are composed of not just chloride but oxide species as well. Three additional components are present in the N 1s spectra and were assigned to N^δ+^, amine, and carbodiimide (Figure 4). Both amines and carbodiimides are known to be present in solid deposits of pMDI.^2^ The N^δ+^ component is indicative of a combination of intermolecular forces between nitrogen-containing groups within the pMDI mixture and/or interfacial interactions between pMDI and the metal surface. Such interactions between MDI and metal surfaces have been identified previously.^13,16^

Chlorine is present across all samples at concentrations in the range of 0.7–3.8 atom %. Chloride and bound organic chlorine atoms have a large difference in binding energy (approximately 2 eV) and can be easily distinguished from one another. All samples have a Cl 2p_3/2_ binding energy of 198.3 eV, which is consistent with chloride species and is assigned as metal chloride and/or surface-bound hydrogen chloride. An additional organic chlorine component with a binding energy of 199.6 eV was identified in 316L-04. This binding energy is consistent with the organic-bound chlorine and is assigned as organochloride reaction products between pMDI and HCl.

The Fe 2p_3/2_ binding energy is consistent with Fe(III) (711.4 eV); however, such clarity is not observed in the Fe 2p satellites between the Fe 2p_3/2_ and Fe 2p_1/2_ peaks, which show evidence for both Fe(II) and Fe(III). As the Fe(II) component will have a lower binding energy, at low concentration, this will merely be present as a broadening of the main Fe 2p components, which may not be evident by inspection. Based on the mixed satellite structure, a mixture of Fe(III) and Fe(II) is assigned on the surface. The total chloride concentration does not account for the concentration of all metal species taking into account the stoichiometry of the proposed chlorides. Based on this, these metallic species are currently assigned as a mixture of metal chloride and oxide species and will be investigated further in the ToF-SIMS analysis discussed below. The binding energies of the Cr 2p and Ni 2p spectra are indicative of Cr(III) and Ni(II). The satellite structure of the Ni 2p spectrum is indicative of NiCl_2_.^17,18^ Both Cr 2p and Ni 2p spectra can be found in the Supporting Information.

Carbon and nitrogen show a trend of decreasing concentration with increasing immersion duration. The iron and chromium show complementary changes of increasing concentration with increasing immersion duration. Except for 316L-02, chlorine also shows an overall trend of increasing concentration with increasing immersion duration. As the carbon and nitrogen are representative of the surface organic material and iron and chromium of the substrate (unless there is a significant migration of organo-metallic species from the interface to the outer surface of the organic layer), this observation indicates a decreased overlayer thickness with increased immersion duration. The observed trend in iron and chromium concentrations assumes that most metal species are located in the interfacial region. As chlorine shows the same trend as iron and chromium, the chloride species are also primarily located in the interfacial region. In addition, 316L-04 presents with the highest C 1s and N 1s concentrations of all samples and no metal photoelectron peaks are present. This suggests that the thickest organic overlayer was deposited from the low concentration solution. To investigate this further, the equivalent overlayer thickness was calculated using the C 1s peak intensity (area).

Equation 1 is a modified version of the Beer–Lambert equation^19^ that can be used to calculate the equivalent overlayer thickness, based on the C 1s intensity of a pMDI layer, where
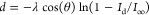
1*d* is the overlayer thickness, λ is the electron attenuation length, θ is the electron take-off angle relative to the sample surface normal, *I*_d_ is the area of the C 1s peak area of the thin layer, and *I*_∞_ is the C 1s peak area of an infinitely thick layer.

The value for *I*_∞_ was obtained from a thick deposit of pure pMDI deposited on a steel substrate. *I*_d_ was calculated from the average C 1s peak intensity from three random analysis areas on the submerged region of each sample. λ was calculated from the average inelastic mean free path of organic materials using the approach of Tanuma et al.^20^ Calculated equivalent overlayer thicknesses and the standard deviations (calculated from three separate measurements) are detailed in Table 2.

**Table 2:** Calculated Equivalent Overlayer Thickness for All Samples

sample	equivalent overlayer thickness nm
316L-01	5.9 ± 0.6
316L-02	6.2 ± 0.9
316L-03	7.0 ± 0.4
316L-04	11.4 ± 1.0

A decrease in surface coverage with increased immersion duration is observed. The calculated thickness does not increase in a monotonic manner, but it is important to remember that this approach provides an equivalent thickness, integrating over an area on the order of 1 mm^2^. The procedure assumes that all carbonaceous species have the same chemistry as pMDI, clearly not the case. However, it provides a clear indication and together with other features of the XPS analysis confirms a trend. This trend is also seen in carbon, nitrogen, iron, and chromium concentrations and the visual analysis of the samples carried out above. This is unexpected as one would predict an increased immersion duration to allow more time for organic material to adhere to the surface; however, this is not the case observed here.

Thus, the principal observations from the XPS analyses are:

- Decreasing overlayer thickness with increased immersion duration.
- Decreasing overlayer thickness with increased solution concentration.
- Identification of metal chloride species, specifically NiCl*_x_* species.

**Figure 5:**
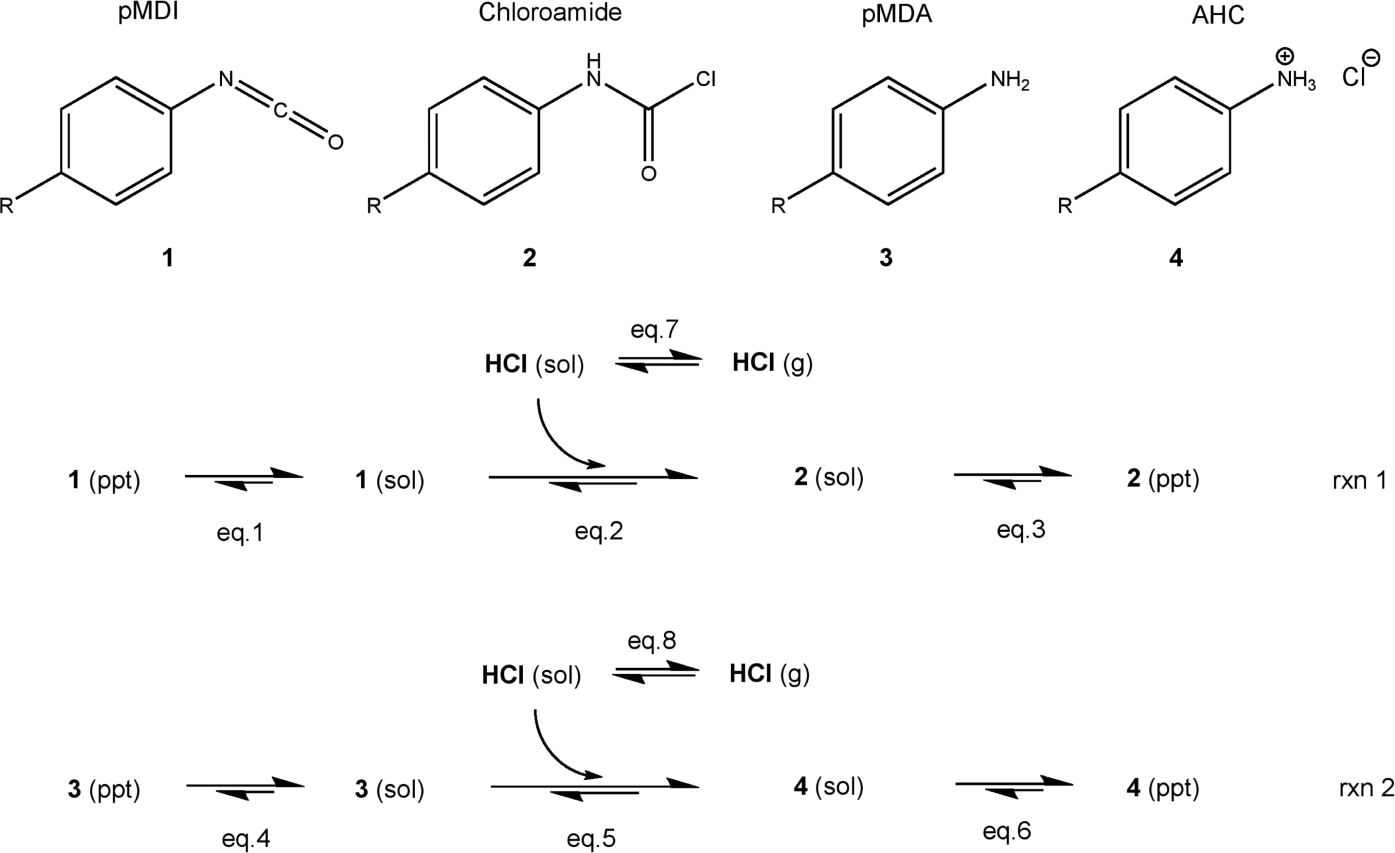
Reaction of pMDI and pMDA with hydrogen chloride. Note eq *x* stands for equilibrium *x*, where *x* = 1–8.

The decreased organic surface concentration with increased immersion duration can be explained by the solubility of the different pMDI derivatives, which will form in this system. Figure 5 shows a reaction mechanism for the reaction of pMDI and polymeric methylene diphenyl diamine (MDA) with hydrogen chloride, forming a chloroamide and an amine hydrochloride (AHC) reaction product, respectively. Note that the chloroamide and AHC reaction products will be referred to as “organochlorides” when they are collectively mentioned from this point forward.

The dielectric constant (κ) can be used to estimate the relative solubility of these species in MCB. Solvents and solutes with smaller differences in κ will have increased solubility. One must, however, treat this correlation with caution. Other chemical properties of the system may have a greater influence over the solubility than the properties governing κ have alone. The ability of solutes to form hydrogen bonds with the solvent, for example, can greatly influence the solubility of a solute in practice. The chemical species considered here are chemically similar, however, and κ should be sufficient to qualitatively identify which species will have the greater solubility in MCB.

The dielectric constants for MCB and pMDI are 5.7 (at 20 °C)^21^ and 7.3 (at 40 °C),^22^ respectively. Although the temperature of these two references for κ differs, they still act as a reliable reference for pMDI having a higher dielectric constant than MCB. The chloroamide group is more polar than the isocyanate group with the addition of the chlorine atom, and therefore the chloroamide will have a larger dielectric constant than its isocyanate counterpart. This same trend will be observed with pMDA and the AHC reaction product. This shows that the chloroamide is less soluble than MDI in MCB, and similarly, AHC is less soluble than pMDA in MCB.

With the differences in solubility of the constituents established, a reaction mechanism can now be proposed, which accounts for the trend of increased overlayer thickness with decreased immersion duration. As described above, the organochlorides will be less soluble in MCB than their isocyanate/amine reactant counterparts. Therefore, the organochlorides will more favorably precipitate out of solution, adhering to the metal surface compared to their reactant counterparts. This is represented by the equilibrium arrows of eqs 1 and 3 and eqs 4 and 6 in Figure 5. The precipitated and dissolved species are denoted by ppt. and sol. in Figure 5, respectively. The organochlorides tend toward the precipitate, and the pMDI and pMDA tend toward the solution.

When HCl is bubbled through the solution for 30 min prior to heating, a portion of it will dissolve in the MCB solution and react with pMDI and pMDA forming their respective organochlorides. Therefore, just before heating, at *t* = 0, we can assume that more of the system will be composed of the organochlorides. At this point, more organic material will favorably precipitate out of solution and adhere to the metal surface leaving a thicker organic overlayer. This accounts for the thicker overlayer observed on 316L-03.

As time progresses, gaseous HCl is slowly released from the system as the chamber is continuously purged with nitrogen. This will shift the equilibrium of HCl toward the gaseous state, removing it from solution. The decrease in HCl_(sol)_ concentration will drive the main reaction (eqs 2 and 5 in Figure 5) toward the reactants, removing the organochlorides from the solution. This decreased organochloride_(sol)_ concentration will have the knock-on effect of promoting the dissolution of the precipitated surface-bound organochlorides, decreasing the overlayer thickness. As pMDI and pMDA have increased solubility over the organochlorides in MCB, less material will precipitate out of solution, adhering to the metal surface. This process results in a decreased overlayer thickness with increased immersion duration.

XPS concentration and equivalent overlayer thickness calculations show 316L-04 to have the thickest overlayer. This suggests a trend of decreasing deposited organic material with increasing solution concentration, another unexpected observation, but one that is accounted for by the proposed reaction mechanism. The 5% v/v solution will exhibit properties (and a dielectric constant) much closer to that of MCB than the 50% v/v solution will. The decreased dielectric constant of the 5% v/v solution will result in the less favorable dissolution of organochlorides than in the 50% v/v solution. Their lower solubility promotes precipitation from solution even further, increasing the overlayer thickness. The organic Cl 2p component in 316L-04 also coincides with this observation. The increased tendency for chloroamide precipitation in low concentration solutions will be associated with the organic Cl 2p component, which is only present in the low concentration sample.

It is important to note, however, that pMDI is a complex mixture of various MDI isomers containing higher homologues and various functional groups such as amine hydrochlorides, carbodiimides, ureas, biurets, and uretonimines. With such complex chemistry, the chemical properties of the system determining solubility will extend further than differences between the isocyanate and chloroamide groups. In addition to organic-metal interactions, solvent–metal and solvent–organic interactions will also play a role in the larger mechanism governing organic deposition. For our investigation, however, we are focused on the chemical properties directly or indirectly affected by the independent variables of the system and how solubility is influenced by these variables. By tightly controlling a small number of independent variables and thoroughly exploring all changes in the dependent variables, a mechanism has been developed, which accounts for the observations made and forms a part of the larger mechanism responsible for organic deposition.

The XPS analysis thus indicates that both surface composition and equivalent overlayer thickness support the trend of reducing overlayer thickness as a function of increased immersion duration or solution concentration. A reaction mechanism has been proposed, which accounts for these observations through the relative solubility of pMDI and its reaction products. The presence of organic chlorine species coincides with the proposed scheme. In the next section, ToF-SIMS analysis of these samples will be presented. The proposed reaction mechanism will be evaluated against the ToF-SIMS analysis. The metal species identified in the XPS analysis will be explored further in the ToF-SIMS analysis.

### ToF-SIMS Analysis

**Figure 6:**
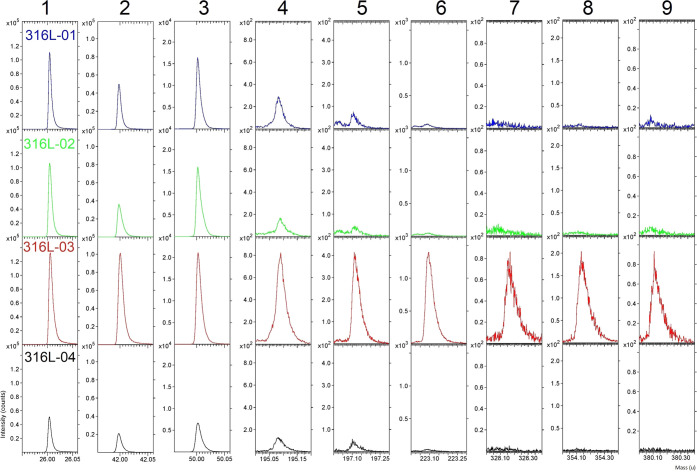
High-resolution negative polarity ToF-SIMS spectra of pMDI characteristic fragment in all samples.

ToF-SIMS analysis of all samples shows specific fragments indicative of pMDI in both positive and negative spectra (Table 3). The Δ parameter is a comparison of the actual mass of an ion recorded under high-spectral-resolution conditions with the theoretical exact mass of the proposed ion and indicates the accuracy of an assigned fragment to the theoretical mass. All fragments have assignments of less than 100 ppm as defined by the Δ parameter, confirming the fidelity of the assignments. The fragmentation yield of pMDI fragments larger than 249 au (molecular ion of MDI) is greater in the negative polarity than in the positive. These larger pMDI characteristic fragments are only present in 316L-03 (Figure 6).

**Table 3:** Table of pMDI Characteristic Fragments in Positive and Negative ToF-SIMS Spectra

positive fragments		negative fragments		
mass (au)	assignment	mass (au)	assignment	reference number
77.045	C_6_H_5_^+^	26.006	CN^–^	(1)
91.056	C_7_H_7_^+^	41.999	NCO^–^	(2)
106.076	C_7_H_8_N^+^	50.003	C_3_N^–^	(3)
132.056	C_8_H_6_NO^+^	195.085^a^	C_13_H_11_N_2_^–^	(4)
165.067	C_13_H_9_^+^	197.114^a^	C_13_H_13_N_2_^–^	(5)
180.083	C_13_H_10_N^+^	223.092^a^	C_14_H_11_N_2_O^–^	(6)
195.089	C_13_H_11_N_2_^+^	328.178^a^	C_21_H_18_N_3_O^–^	(7)
197.117	C_13_H_13_N_2_^+^	354.156^a^	C_22_H_16_N_3_O_2_^–^	(8)
223.097^a^	C_14_H_11_N_2_O^+^	380.127^a^	C_23_H_14_N_3_O_3_^–^	(9)

a Indicates that the fragment was only strongly observed in 316L-03. The reference number of column 5 identifies the high-resolution ToF-SIMS spectra of Figure 7.

The two most intense fragments characteristic of pMDI in the positive spectra are those at *m*/*z* = 106 and 132 au (Figure 7). These fragments are indicative of reacted and unreacted isocyanate groups, respectively, and can be used as an indication of the degree of reacted isocyanate groups of the pMDI deposited on the surface.^15^Table 4 compares the intensity ratio of the 106:132 fragments. The ratio is much larger in 316L-01 and 316L-02 than in 316L-03. This is expected as more isocyanate groups will have reacted forming various amines, ureas, carbodiimides, and uretonimines with increased immersion duration at elevated temperatures. The ratio is also larger in 316L-04, treated in the more dilute solution than in 316L-03 even though both samples were immersed for 5 min.

**Figure 7:**
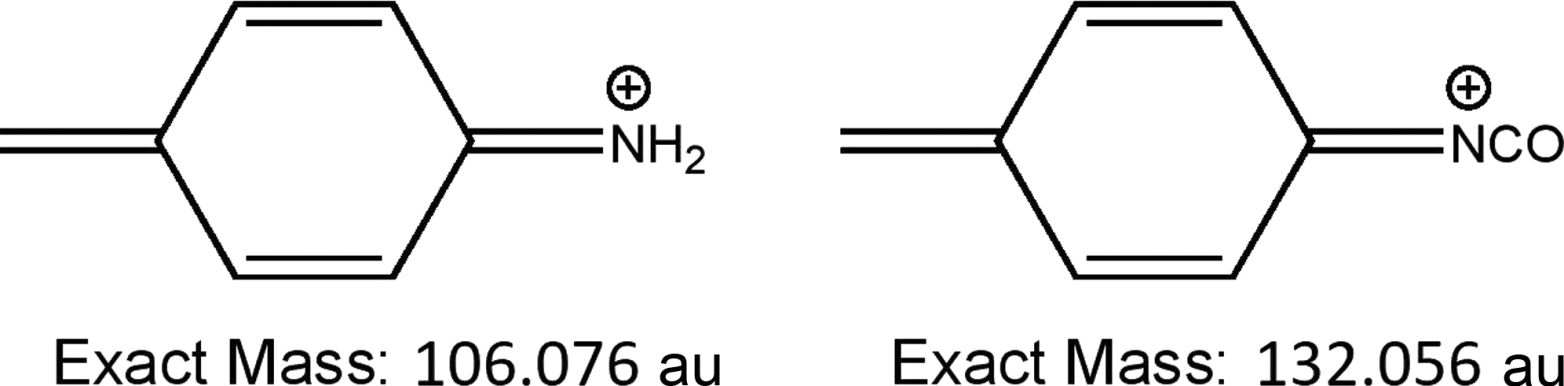
Structure of 106 and 132 pMDI characteristic fragments in the positive SIMS analysis.

**Table 4:** Ratio of 106:132 Fragments in the Positive SIMS Spectra of the 316L Samples

sample	106:132 ratio
316L-01	4.1
316L-02	4.1
316L-03	2.3
316L-04	3.8
pure pMDI	1.0

Of the transition metals, Fe^+^, Cr^+^, Ni^+^, and Cu^+^ fragments were identified in the positive SIMS spectra. The intensity of the Fe^+^ and Ni^+^ fragments follows the trend of increased intensity with increased immersion duration. This is in agreement with equivalent overlayer thickness calculations from the C 1s peak intensity in the XPS analysis. The intensity of the Cu^+^ fragment is approximately an order of magnitude lower than that of Fe^+^, Cr^+^, and Ni^+^ fragments in all spectra except 316L-04 where they are approximately equal. The absence of any copper photoelectron peaks in the XPS analysis and its low intensity in the ToF-SIMS analysis suggests that it is present in trace concentrations and could be present as trace species in the steel substrate.

The negative SIMS analysis reveals two regions with well-defined clusters of fragments. These range from 120 to 140 and 150 to 170 au. In the XPS analysis, various metal oxides and/or chlorides were proposed to be present on the surface. The approximate mass of these clusters is consistent with a combination of various first-row transition-metal oxides and chlorides.

**Figure 8:**
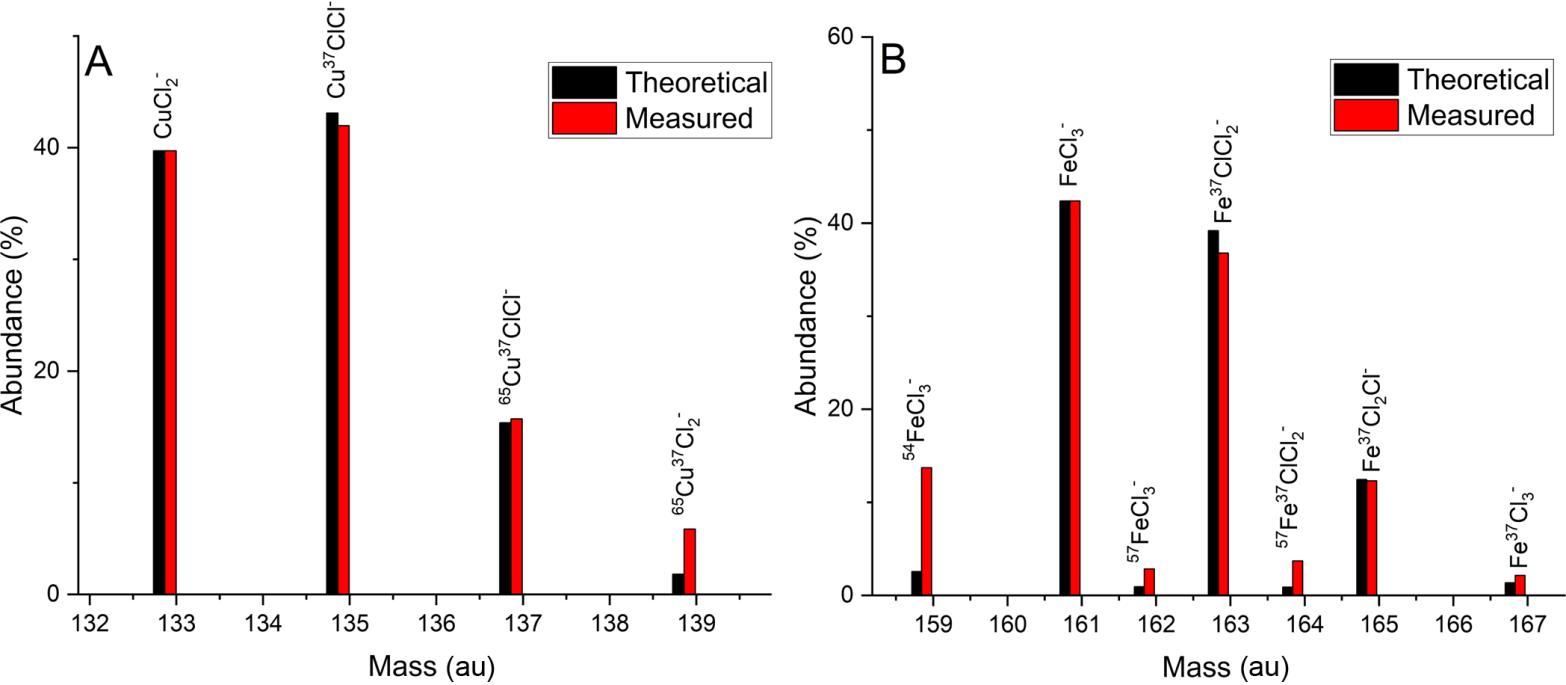
Theoretical and measured intensities of molecular isotopes of (A) CuCl_2_ and (B) FeCl_3_ from the negative SIMS spectrum of 316L-04.

The ToF-SIMS spectrum 316L-04 shows two clusters of fragments indicative of CuCl_2_^–^ and FeCl_3_^–^. The isotopic match value is used to indicate how closely the theoretical molecular isotope intensities match with the measured intensity of the spectrum. The calculated (theoretical) value indicates the theoretical peak intensity of all molecular isotopes at a specific molecular mass. This is measured against the measured intensity to determine how well any combination of molecules and their isotopes fits with the measured spectrum. The peaks have accuracies of better than 40 ppm and theoretical molecular intensities in keeping with measured intensities with isotopic match values of 94.8 and 91.9%, respectively (Figure 8), confirming their identity. No other compound met all of these observations as closely as CuCl_2_ and FeCl_3_. 316L-03 showed similar results with well-defined clusters of peaks representative of CuCl_2_ and FeCl_3_. Similar results were observed on samples 316L-01 and -02.

The corresponding peaks in the spectra of 316L-01 were assigned as the isotopes of CuCl_2_^–^ and FeCl_3_^–^. These two species alone were not enough to account for all significant peaks observed from 115 to 140 au on 316L-01, however. Care must be taken when assigning the rest of these peaks ensuring the following criteria are met:

- Theoretical intensity based on isotope ratios aligns with the measured intensities.
- Mass deviation of theoretical mass from the measured mass is within reasonable bounds (<100 ppm).
- The proposed fragment is feasible from a fragmentation pathway standpoint.

**Figure 9:**
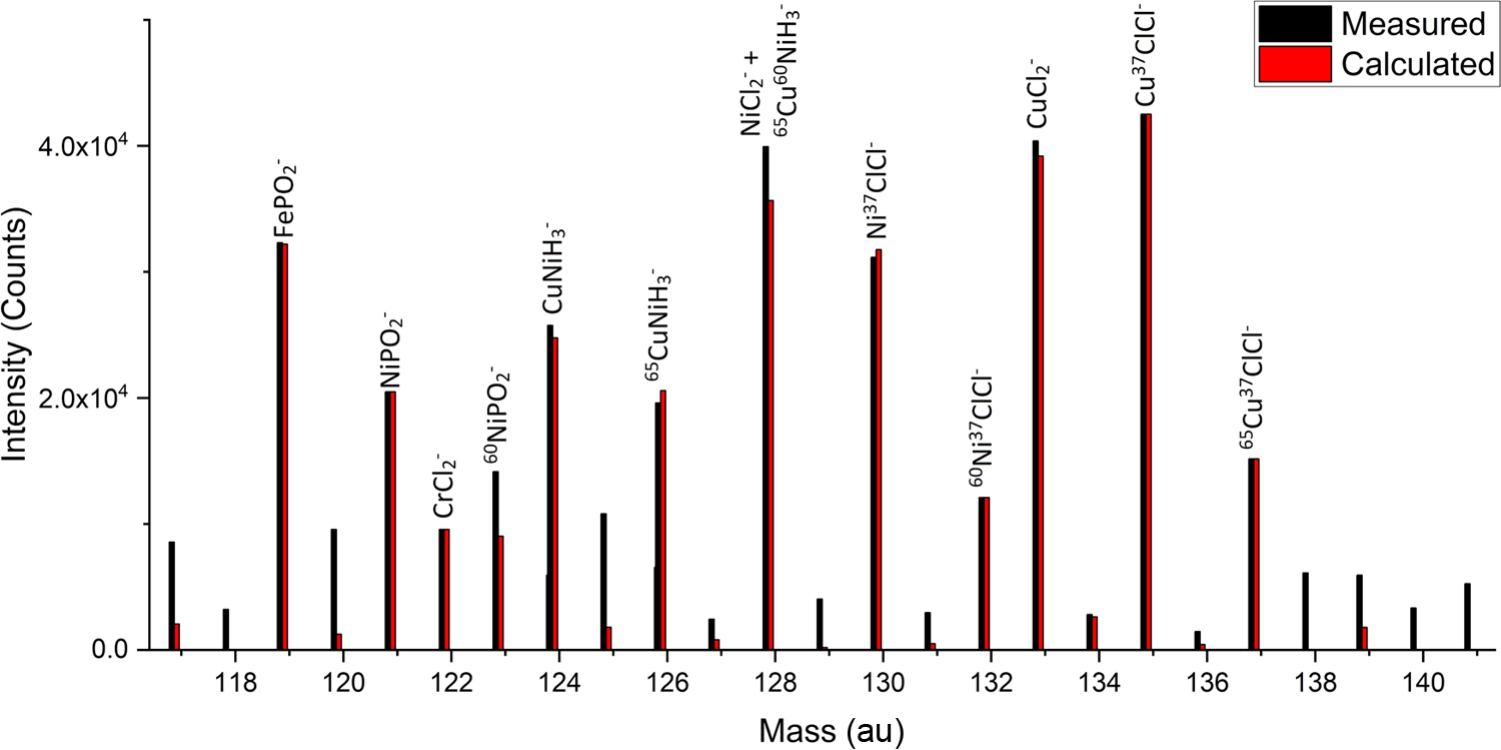
Measured vs calculated (theoretical) intensity for proposed SIMS fragments for the negative spectrum of 316L-01. Mass range 115–140 au.

An example of the procedure used to assign the clusters of peaks following the above criteria is detailed in the Supporting Information. The combination of FePO_2_^–^, NiPO_2_^–^, CuNiH_3_^–^, NiCl_2_^–^, CuCl_2_^–^ (including their molecular isotopes), and either CrCl_2_^–^ or CuNiH^–^ accounts for the intensity of all significant peaks for the 115–140 au cluster (Figure 9). Some of the less significant peaks are not fully accounted for by these species; however, identifying these main species informs the surface chemistry sufficiently.

**Figure 10:**
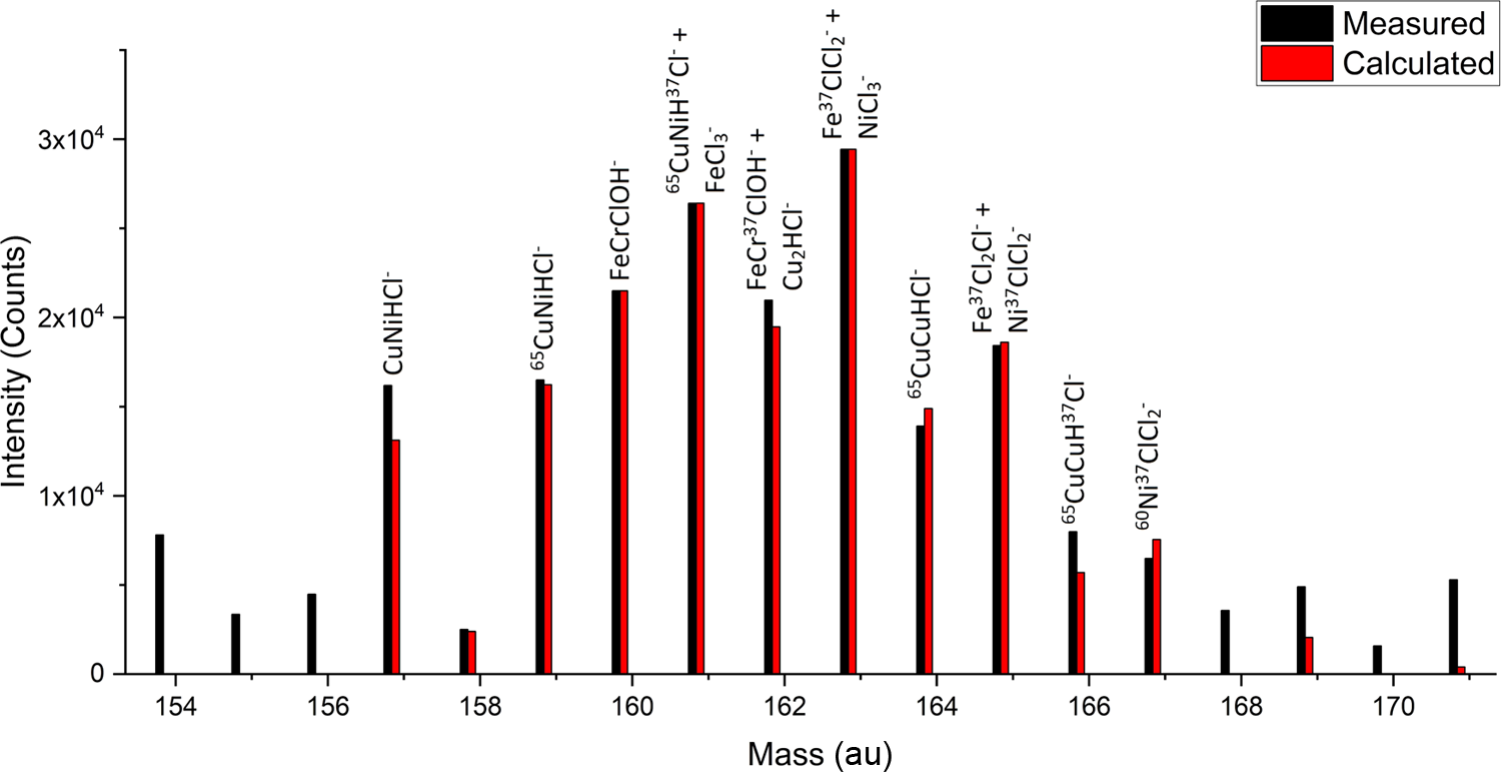
Measured vs calculated (theoretical) intensity for proposed SIMS fragments for the negative spectrum of 316L-01. Mass range 155–170 au.

CrFeClOH^–^, FeCl_3_^–^, Cu_2_HCl^–^, and NiCl_3_^–^ were identified in the 150–170 au cluster by following the same procedure in assessing the plausibility of each assignment. The combination of their theoretical molecular isotope ratios fits with the measured intensity well, and all have deviations of less than 100 ppm, confirming the fidelity of their assignment (Figure 10). Based on additional characteristic fragments not listed here, the source of FePO_2_ and NiPO_2_ phosphate species was identified as a phosphorous antioxidant additive from an unspecified source.

A wide range of metal chlorides and oxides were identified from the negative SIMS analysis of these samples. The presence of iron, chromium, and nickel chlorides agrees with observations made in the XPS analysis. Although no copper was identified in the XPS analysis, the greater sensitivity of ToF-SIMS will have detected trace amounts of copper chloride, which were not detectable with XPS.

Numerous species that indicate hydrogen chloride attack on the metal surface have been identified through the combination of XPS and ToF-SIMS analyses. Removal of the passive film under conditions where oxygen is not readily available to repassivate the metal surface will increase the susceptibility of the metal to corrosive attack. Removal of the passive film will leave behind a more reactive surface, which will have increased susceptibility to pMDI adhesion. Only iron chloride is present across all four samples. With the thicker layers formed on 316L-03 and 316L-04, this suggests that iron is migrating through the organic overlayer during its formation. Interactions between MDI and iron-containing metal surfaces have been observed previously.^9,13,14^ It is proposed that these reacted iron species migrate through the organic material, depleting the metal surface of iron.

The increased 106:132 ratio of 316L-04 over 316L-03 can be explained by the increased tendency for the organochlorides to precipitate from the low concentration solution. The 106 fragment is indicative of the organochlorides, and therefore the ratio will increase as more adheres to the surface. The 106:132 ratio is consistent in predicting increased reacted isocyanate with increased immersion duration. It is important to note, however, that the 106 fragment is not only indicative of reacted isocyanate groups (forming various amines, ureas, carbodiimides, etc.) but of interfacial bonds between pMDI and the substrate through nitrogen–metal bonds as well. Although an increase in the 106:132 ratio correlates with increased immersion duration, this does not confirm that increased reacted isocyanate groups are the only cause of this correlation. Interfacial bonds may also be involved in the observed correlation.

In recent work from this laboratory by Bañuls-Ciscar et al., several organo-metallic fragment ions, in both the positive and negative SIMS spectra, were identified.^14^ These were ascribed to the interaction of pMDI with the alloy steel (Fe_0.8_Cr_0.2_) substrate. These ions were used as a basis of a thorough search for evidence of similar compound formation in the current study. None were observed in the positive SIMS spectra. Fragment peaks indicative of these organo-metallic ions were observed in some negative spectra. The samples with thinner organic overlayers generally presented higher intensities. However, when compared against reference spectra of the metal substrate and pure pMDI, a definitive conclusion on their presence cannot be reliably made. In the case of the thicker organic overlayers, the analysis depth of SIMS does not sufficiently extend to such depths without resorting to cluster ion beam profiling, as exemplified by the work of Bañuls-Ciscar et al. The stronger association of the organo-metallic fragments with thinner organic overlayers is consistent with them being present at the interfacial region but in a rather low concentration. It is important to remember, however, that the conditions presented here differ from those studied by Bañuls-Ciscar et al. Specifically, the HCl gas treatment could impact the bonding mechanism at the interface leading to different interfacial chemistry.

The absence of any high mass pMDI characteristic fragments in the negative spectrum of 316L-04 can be explained by the free-energy difference of dissolved and precipitated organic species in the system. Shorter-chain pMDI species, specifically 4,4′-MDI, have a larger free-energy difference between their dissolved and precipitated states than longer-chain pMDI species. Following the system equilibria established from Figure 5, the difference in free energy between shorter- and longer-chain pMDI species will be greater in the 5% v/v than in the 50% v/v solution. Therefore, the precipitation of shorter-chain species will be favored more in the 5% v/v compared to that in the 50% v/v solution and this is observed through the lack of longer-chain pMDI fragments in the negative SIMS spectrum of 316L-04.

**Figure 11:**
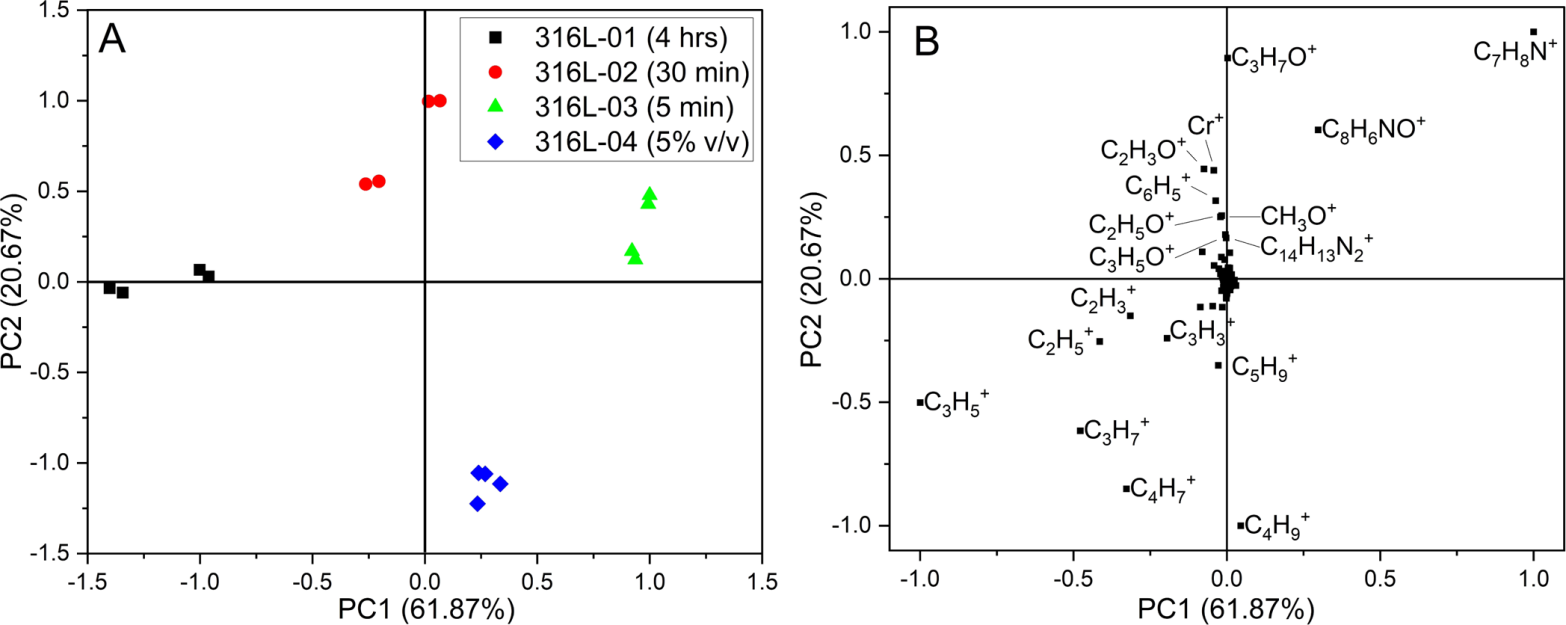
Positive peak list PCA: (A) score biplot and (B) loading biplot for PC1 vs PC2. The explained variance for the total data set for each PC is given in parentheses.

Principal component analysis (PCA) was employed to identify patterns in the ToF-SIMS spectra among the samples. The peak list selection procedure for PCA analysis is detailed in the Supporting Information. The positive peak list showed good clustering of repeat measurements on each sample and clear separation between the different samples on the score biplot (Figure 11). A variance greater than 95% was achieved with three principal components (PCs) for the positive data set.

Analysis of the score plots shows that PC1 separated the samples by immersion duration and PC2 by solution concentration. PC3 does not show an obvious trend in the sample preparation conditions, differentiating 316L-02/04 from 316L-01/03 and will not be discussed here. The positive data set score biplot for PC1 vs PC3 and the corresponding loading biplot can be found in the Supporting Information.

From the analysis of the loading biplot, long immersion durations are associated with C*_x_*H*_y_*^+^ fragments and short immersion durations with pMDI characteristic fragments. Low solution concentrations are associated with C*_x_*H*_y_*^+^ fragments and high solution concentrations are associated with Cr^+^, C*_x_*H*_y_*O*_z_*, and pMDI characteristic fragments.

**Figure 12:**
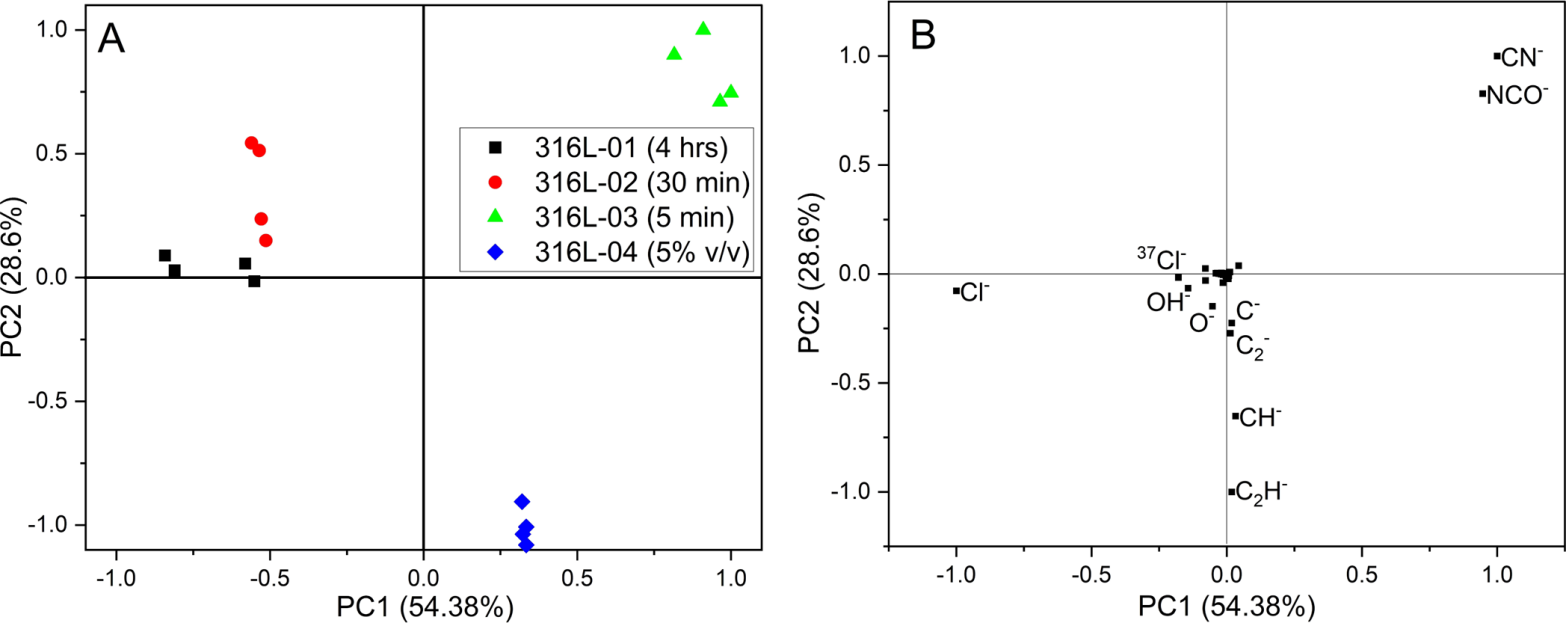
Negative peak list PCA: (A) score biplot and (B) loading biplot for PC1 vs PC2. The explained variance for the total data set for each PC is given in parentheses.

The PCA analysis of the negative data set showed good clustering of repeat measurements and clear separation between the different samples on the score plot (Figure 12). Three PCs achieved a variance of 94.4%. For clarity of discussion, the first three PCs will be considered to have a variance great enough to represent the data set. Analysis of the score plots shows that PC1 separated the samples by immersion duration and PC2 by solution concentration. PC3 does not show an obvious trend in the sample preparation conditions and will not be discussed here. The negative data set score biplot for PC1 vs PC3 and the corresponding loading biplot can be found in the Supporting Information. From the analysis of the loading biplot, long immersion durations are associated with Cl^–^ and OH^–^ and short immersion durations are associated with pMDI characteristic fragments. All metal chloride species have a weak association with long immersion durations but have not been labeled in Figure 12 for figure clarity. Low solution concentrations are associated with C*_x_*H*_y_*^–^ fragments and high solution concentrations are associated with pMDI characteristic fragments.

Although 316L-04 has a weak association with pMDI characteristic fragments in PC1 in both polarities, one would expect a strong association considering 316L-04 has one of the thickest organic overlayers. All other observations are in agreement with the XPS analysis, however. The association of short immersion durations with pMDI characteristic fragments is consistent with a decreased overlayer thickness as a function of increased immersion duration. The association of Cl^–^ and metal chloride species with long immersion durations suggests that these species are primarily located in the interfacial region. The weak association of the metal chloride species can be accounted for by their lower peak intensities compared to lower *m*/*z* fragments. The detection efficiency of ToF-SIMS detectors generally decreases with increased fragment mass.^23^ This is a limitation of PCA analysis of ToF-SIMS data as it skews the variance contribution of higher mass fragments resulting in a lower loading contribution from higher mass fragments with the same relevance as lower mass fragments. Implementation of correlation loadings has been shown to compensate for this limitation but is not a feature available in simMVA.^24^

Low solution concentrations show a strong association with C*_x_*H*_y_* fragments in both polarities. The absence of a strong association with pMDI characteristic fragments is accounted for as a result of solubility differences found in the system. The preferential precipitation of shorter-chain pMDI molecules in the 5% v/v solution will alter the overall chemical structure of the material deposited. Under Bi_3_^+^ ion bombardment in the ToF-SIMS analysis, more fragments indicative of pMDI such as the 106 and 132 au fragments (Figure 7) are likely to be yielded from longer pMDI molecules than from shorter pMDI molecules, including 4,4′-MDI. This is because the shorter-chain pMDI molecules are a small number of fragmentations away from the smallest characteristic fragments such as 106 and 132 au. Larger pMDI molecules can undergo multiple fragmentations and still yield fragments as large or larger than the 106 and 132 au fragments and therefore will have an increased probability of yielding them. One must, however, remember the quantitative limitations of the ToF-SIMS technique. The presence of any specific fragments is linked to various parameters such as concentration, inherent stability, network cross-linking, and probability of formation of the said fragment. The XPS analysis of these samples confirms a thick overlayer on 316L-04.

## Conclusions

In this work, the chemical characteristics of 316L steel samples exposed to conditions related to those found in the pMDI manufacture have been fully characterized. Both XPS and ToF-SIMS analyses showed strong evidence for the presence of pMDI on the surface. An increase in the equivalent overlayer thickness was observed with decreased immersion durations and pMDI solution concentrations. A reaction mechanism was proposed, which accounts for these observed trends in the overlayer thickness. This indicated that the mechanism governing the initial stages of fouling is strongly influenced by the presence of organochloride species and their relative solubilities. Metal chlorides and surface-bound hydrogen chloride were shown to be present, with most species located at the metal-organic interface.

The ratio of the 106:132 au mass fragments indicates a trend of a greater proportion of reacted isocyanate groups in pMDI as a function of increasing immersion time. The 106:132 au ratio is also in agreement with the proposed reaction mechanism. Various metal oxides were identified, and iron chloride was proposed to migrate through the deposited organic layer, a phenomenon that has been observed previously.^25^

In relation to previous work undertaken with a binary Fe/Cr alloy and various solutions of pMDI, evidence of specific interaction between the metal and pMDI was inconclusive.^14^ This is an important observation concerning the fouling of the MDI process plant, highlighting the significance of the process chemistry involved.

The PCA analysis has informed and supported the conclusions drawn from the XPS and ToF-SIMS analyses. Both polarity data sets separated samples based on immersion duration and solution concentration. pMDI characteristic fragments were associated with short immersion durations. This agrees with the proposed reaction mechanism from the observations made in the XPS and ToF-SIMS analyses. Fragments indicative of metal chlorides were associated with thinner organic overlayers, suggesting that these metal chlorides are mainly present at the metal/organic interface. The weak association of metal chloride fragments can be explained by their lower intensity compared to other lower *m*/*z* fragments that have a similar relevance but higher loading value due to their higher intensity.

Future work will explore the effect HCl has on the composition of organic material and stainless steel surfaces, informing the use of corrosion-resistant materials for engineering and operating applications in MDI production. This work has focused on replicating the initial fouling conditions found in the production plant. Future experiments will aim to bridge these initial conditions to the large-scale fouling observed in the production plant. In parallel, a detailed investigation of Duplex stainless steel (using samples exposed to the same conditions as the AISI 316L considered in this paper) will be undertaken.

## Materials and Methods

### Sample Preparation

Coupons of 50 × 20 mm^2^ were cut from a 2 mm thick 316L stainless steel (supplied by Goodfellow Cambridge Ltd., U.K.). Three millimeter holes were drilled through the top center of the coupons, and sample identification codes were engraved onto the back of each sample. Samples were cleaned in separate ultrasonic baths of acetone (supplied by Sigma-Aldrich) and hexane (supplied by Fisher Scientific) for 30 s each, allowing time for drying in between cleaning. One side of the coupons was then UV–ozone-cleaned (Hitachi High-Technologies, Zone Cleaner) for 20 min. All samples were then placed in glass vials topped with aluminum foil.

**Figure 13:**
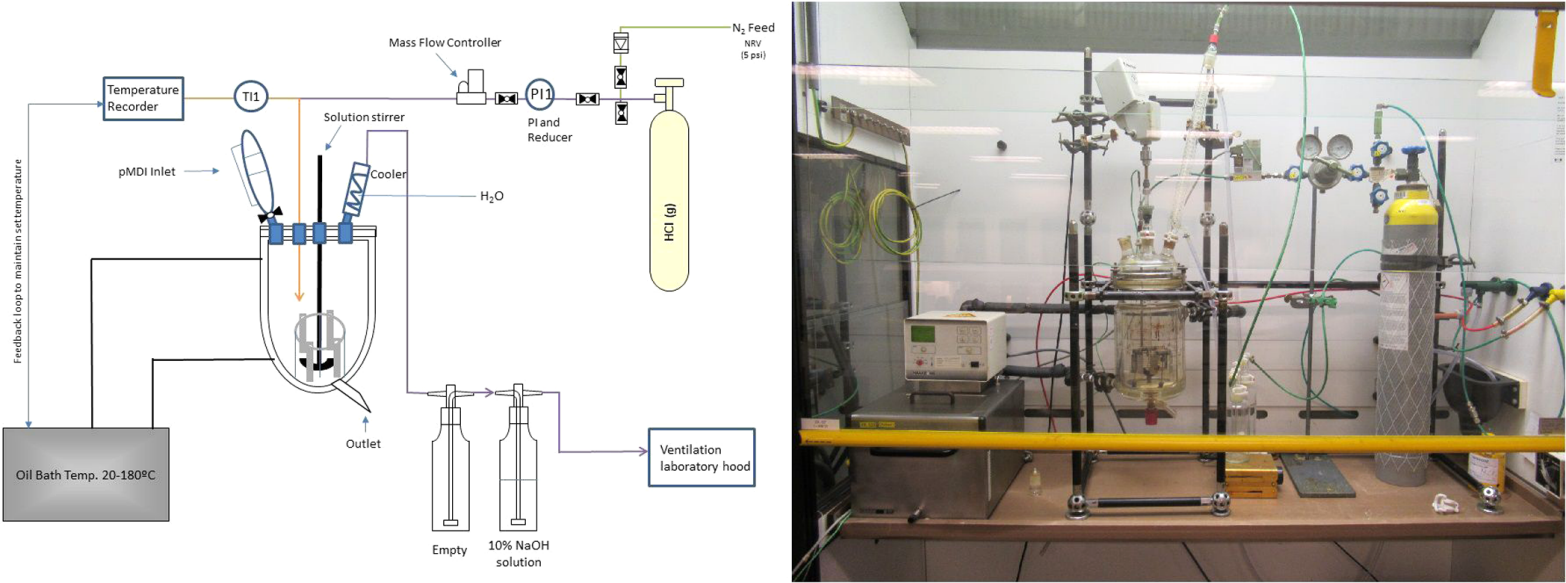
Schematic and image of fouling apparatus.

A schematic and an image of the fouling apparatus used for the following sample preparation are presented in Figure 13. Two solutions of 50 and 5% v/v of pMDI in monochlorobenzene (MCB, supplied by Merck) were prepared. Solid amine hydrochloride (AHC) (5 mol % of AHC to pMDI; see Figure 5) was added to the bottom of the reactor vessel. The coupons were suspended from a stainless steel ring assembly, which was placed on the floor of the reactor. All coupons were positioned so that their UV–ozone-cleaned side faced toward the center of the reactor. Approximately 1 L of either 5 or 50% v/v solutions of pMDI in MCB was added to the reaction chamber, submerging the bottom half of the coupons. The 50% v/v solution was chosen as a representative crude MDI concentration found in the early stages of the separation process. The 5% v/v solution was chosen with the expectation that there would be a direct correlation between the overlayer thickness and solution concentration. Thus, the 5% v/v solution was chosen to ensure that samples were prepared where the organic layer deposited was thin enough for interfacial analysis with XPS and ToF-SIMS without the use of destructive depth profiling.

A thermocouple, a stirrer, and a gas inlet were inserted into the solution, and the chamber was sealed shut. The stirrer was turned on, and hydrogen chloride was bubbled through the gas inlet into the solution for 30 min at a flow rate of 100 mL min^–1^. Afterward, the gas inlet was purged with nitrogen and heating of the reaction chamber started. The nitrogen purge was maintained for the rest of the sample preparation procedure. The reaction chamber was heated to 125 °C after which it was held at this temperature for a time as shown in Table 5, which defines the four experimental runs undertaken and relative pMDI concentrations and exposure times.

**Table 5:** Sample Codes for Immersion Durations and pMDI Concentrations

sample code	immersion duration (min)	pMDI concentration (% v/v)
316L-01	240	50
316L-02	30	50
316L-03	5	50
316L-04	5	5

After the required time, heating was stopped, and the chamber was allowed to cool. Once the chamber reached 70 °C, the nitrogen purge was stopped and the solution was drained from the bottom of the chamber. The chamber was then unsealed, and the samples were removed. All samples were rinsed with MCB and placed in glass vials sealed with aluminum foil prior to analysis. All samples were prepared in triplicate. Following rinsing, but prior to surface analysis, a photographic record was made of all samples using a Canon DSLR camera.

Although phosgene is normally present in the first separation stages, it was specifically excluded from the experimental procedure described here. Principally, this was done to increase the rate of fouling as phosgene is used in the commercial production process to react competitively with methylene diphenyl diamine (MDA), forming pMDI. Without phosgene, MDA reacts with pMDI forming urea groups, which are known to lead to increased fouling in plant conditions.^2^ The reactions between MDI and various species including phosgene and MDA are detailed elsewhere.^2,3^ HCl is present during the first separation stages in MDI production and increases corrosion and potentially etching of the stainless steel surface, which can result in greater adhesion of organic species. Because of this, HCl was used in keeping with the aim of replicating initial plant conditions by accelerating fouling on the lab scale. AHC is present during the separation stages of MDI production and reacts with MDI forming urea species; 5 mol % was added as an overestimation of AHC present during separation stages in MDI production to increase fouling rate while replicating plant conditions. Coupons of clean Duplex stainless steel and tantalum were also placed in this solution but will not be discussed in this paper.

### X-ray Photoelectron Spectroscopy

XPS was used to study the surface and interfacial chemistry of all samples using a K-Alpha^+^ X-ray photoelectron spectrometer (Thermo Scientific, East Grinstead, U.K.). The spectrometer was operated in the constant analyzer energy mode. XPS survey and high-resolution spectra were collected with a pass energy of 200 and 50 eV and a step size of 0.4 and 0.1 eV, respectively. A monochromated Al Kα X-ray source was used with a radial spot size of 400 μm. The C 1s peak was used as the reference peak for charge correction for all spectra acquired. Charge compensation was achieved using an electron flood gun with a filament current of 300 μA and an accelerating voltage of 0.05 V. Subsequent data processing was carried out using the manufacturer’s Avantage v5.890 software. Peak areas of the high-resolution spectra were employed for quantification following the removal of a Shirley (S-type) background. For all peak fitting, a 30% Lorentzian–Voigt function was used.

### Time-of-Flight Secondary Ion Mass Spectrometry

Surfaces were analyzed with the TOF.SIMS 5 (ION-TOF GmbH, Münster, Germany) instrument. Static SIMS conditions (ion dose <10^13^ ions cm^–2^ analysis^–1^) were achieved using a 25 keV Bi_3_^+^ primary ion beam rastered over an area of 100 × 100 μm^2^, with a 9.0 keV reflectron extractor voltage. Both positive and negative SIMS spectra were acquired in the high-spectral-resolution (high-current-bunched mode) mode over a mass range of 1–800 au. Spectral acquisition and processing were achieved using ION-TOF GmbH SurfaceLab v6.4 software.

ToF-SIMS spectra contain a vast amount of information. Spectra are commonly recorded over mass ranges of hundreds to thousands of mass units (1–800 au in the current work), with each nominal mass interval potentially containing multiple fragments. This makes univariate analysis of ToF-SIMS spectra challenging when comparing a few fragments and practically impossible for all fragments present. Multivariate analysis methods are commonly employed in the analysis of ToF-SIMS spectra to enable the analysis of such large data sets. Principal component analysis is the most commonly employed multivariate analysis method for processing ToF-SIMS data. Here, the MATLAB-based software package simsMVA has been employed in the analysis of ToF-SIMS data sets.^26^

## Supporting Information Available

Further information regarding the polymerization chemistry of MDI; Cr 2p and Ni 2p XPS spectra from 316L-01; detailed description of the peak selection procedure employed for ToF-SIMS PCA analysis and metal fragment identification; and additional scores and loading PCA plots for PC1 vs PC3 for all samples (PDF)
