# Supplementary material for: Interfacial Chemistry Investigation of Initial Fouling Conditions in Isocyanate Production: The Antifouling Performance of AISI 316L Stainless Steel
Source: ACS Omega. 2021 Oct 1;6(40):25950–63. doi: 10.1021/acsomega.1c02711 (PMC8515391; doi:10.1021/acsomega.1c02711)
Supplement: Supplementary file 1 — ao1c02711_si_001.pdf [file ao1c02711_si_001.pdf]

An interfacial chemistry investigation of initial fouling conditions in isocyanate production: The anti-fouling performance of AISI 316L stainless steel

Supporting Information

Clayton Bevas*[1], Marie-Laure Abel[1], Ivo Jacobs[2], Karin van Oudgaarden[2], John F. Watts[1]

Department of Mechanical Engineering Sciences

[1]University of Surrey, Guildford, Surrey GU2 7XH, UK

[2]Huntsman Polyurethanes, Global Excellence Team, Rotterdam, The Netherlands

*c.j.bevas@surrey.ac.uk

The reactivity of isocyanates can be explained by their multiple resonance structures and undergo a variety of different reactions with various species. A more detail review of isocyanate chemistry can be found elsewhere. [The Polyurethanes Book, Randall, David; Lee, Steve] A brief of the reactions most relevant to MDI polymerisation is provided below.

Isocyanates can react together across the C=N bond in a [2+2] cyclo-addition reaction forming 4-membered ring dimers (**Error! Reference source not found.**). Diisocyanates can form polymeric dimers which are insoluble crystalline polymers. Isocyanate dimer formation mainly occurs in aromatic isocyanates and readily occurs in MDI, even at room temperature.

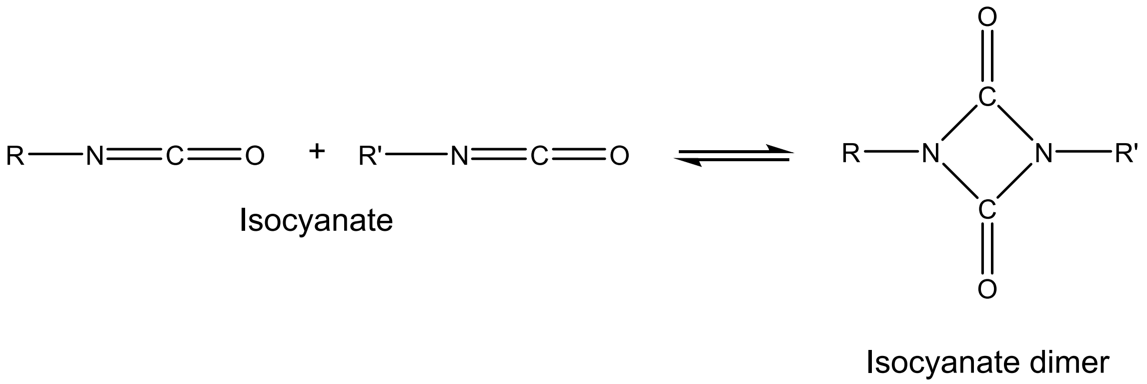

Figure S1 Isocyanate reaction with isocyanate (dimer formation)

In addition to dimers, three equivalents of isocyanate can react to form six-membered ring trimers (**Error! Reference source not found.**). Trimer formation is highly exothermic and continues until all NCO groups have reacted. Both aliphatic and aromatic isocyanates form trimers and under certain conditions can form polytrimers. Trimer formation can be an undesirable side product in MDI production; however, it can also be used to introduce branching and polymeric cross-linking in polyurethane formation. This is particularly useful in the formation of rigid foams where their cross-linked structure contributes to their heat resistance. Trimerisation is an irreversible reaction unlike dimerisation and the resultant trimers are thermally stable up to 270 °C.

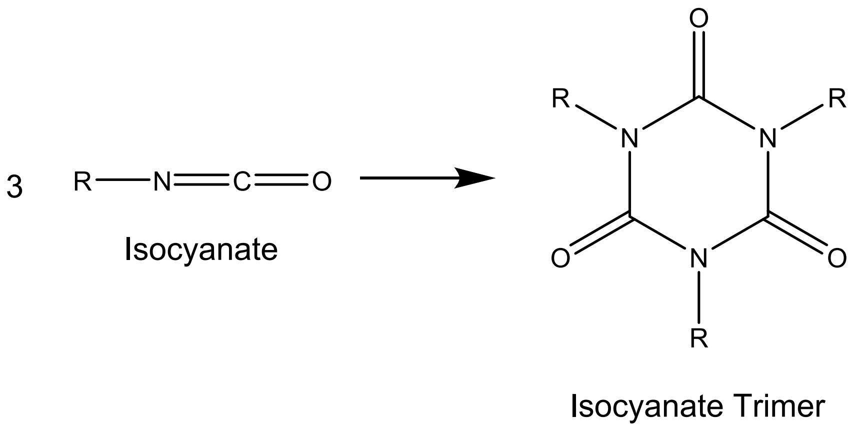

Figure S2 Isocyanate reaction with isocyanate (trimer formation)

As well as dimer/trimerisation, isocyanates readily react to form carbodiimides at temperatures above 180 °C. Carbodiimides can react further with a third equivalent of isocyanate to form uretonimines (**Error! Reference source not found.**). Carbodiimide formation is an irreversible reaction, however,

uretonimine formation is reversible, favouring carbodiimides at high temperatures and uretonimines at lower temperatures. As discussed for biurets and allophanates above, this reversible reaction can be used to re-distribute different carbodiimide and uretonimine groups across the polymer system and/or increase cross-linking.

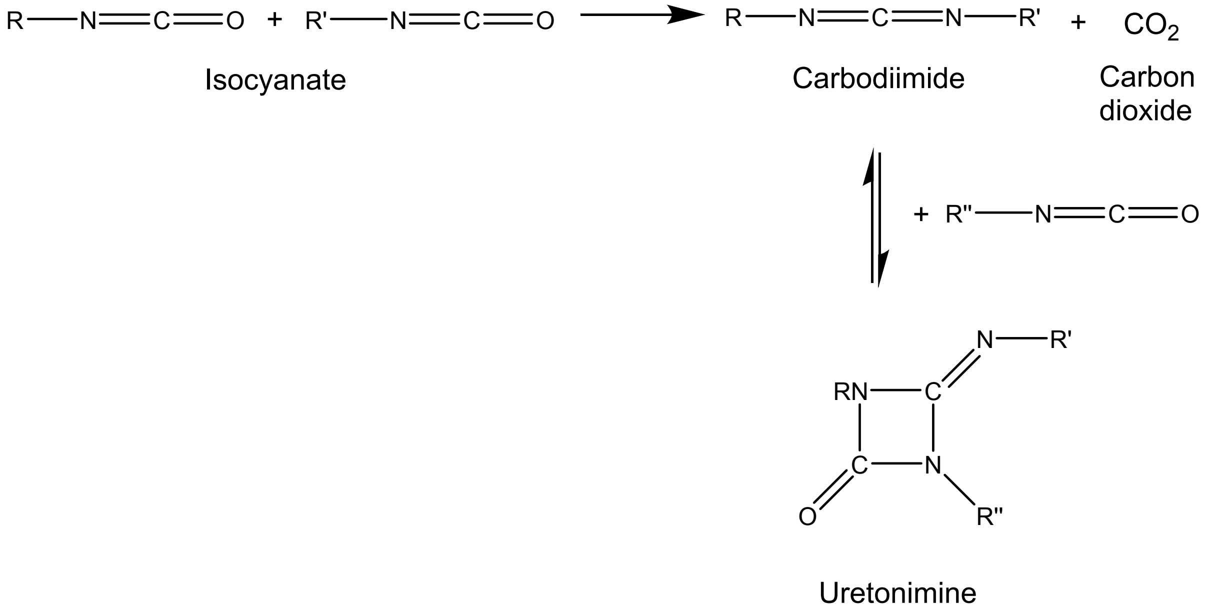

Figure S3 Isocyanate reaction with isocyanate (carbodiimide and uretonimine formation)

Data availability statement. Additional XPS and ToF-SIMS spectra and raw data are available from the corresponding author on reasonable request.

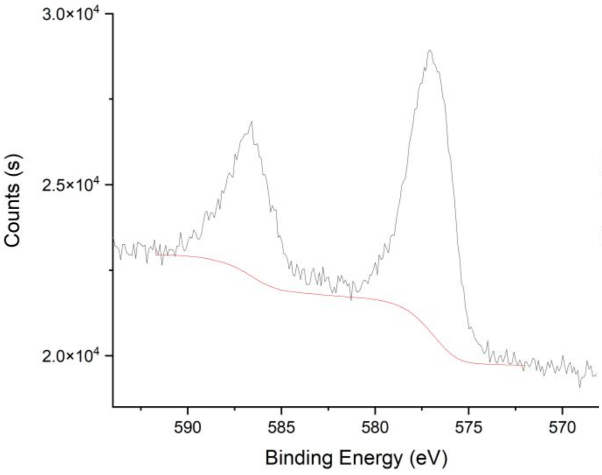

Figure S4 Cr2p XPS spectrum of 316L-01

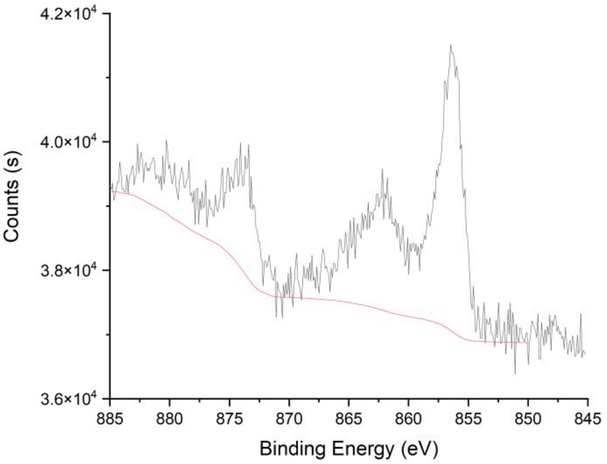

Figure S5 Ni2p XPS spectrum of 316L-02

<u>Peak List Selection</u>

Due to their various isotopes, first-row transition metal oxides and chlorides have multiple molecular isotopes with the same nominal mass. The difference in mass of many of these same nominal mass molecular isotopes is considerably lower than the mass resolution of the TOF.SIMS 5 machine used here. Fragment assignment was achieved using the recalculate function in *SurfaceLab 6*. This function produces a list of molecules with similar masses to the measured peak mass restricted to a combination of atoms from a user input list. The user input list contained the atoms H, C, N, O, Cl, Cr,

Fe, Ni and Cu. The recalculate function also provides an "explained" value from 0 – 100% for each proposed assignment. The explained value gives the user an indication of the plausibility of an assignment based on the spectrum's fit with said fragment's molecular isotopes. If the theoretical intensity (based on natural abundance of the elements in the propose fragment) of the molecular isotope is greater than the measured intensity, then the explained value will decrease. If the theoretical intensity is less than the measured intensity, then the explained value will still be 100% as the theoretical intensity is still be accounted for by the measured intensity.

Another approach to producing a list of potential assignments is using the check function in SurfaceLab *v6.4* which compares the measured mass against a database of known fragments rather than a list of all theoretical combinations of atoms and isotopes. Using the recalculate function produces a larger list of potential fragments compared to the check function, however, it is a more thorough method for examining potential assignments.

All peaks with a deviation of < ± 150 ppm were examined as potential assignments. If a potential assignment meets the following five criteria it is assigned with confidence.

1. It has a deviation of less than 100 ppm for all molecular isotopes.
2. It has an explained value of > 90%.
3. There is good agreement between the theoretical and measured peak intensities (including minimal remaining measured counts not accounted for by the theoretical intensity).
4. It's main molecular isotopes (more than 10% abundance) cannot be accounted for by other plausible molecular isotopes.
5. It makes plausible spectrometric sense based on the known chemistry of the system.

If criteria 2 above is not precisely met and there is significant remaining measured intensity after accounting for the theoretical molecular isotope, then other plausible molecular isotopes were examined. There was a larger number of significant fragments present from 120-170 u on 316L-01 and 316L-02 compared to 316L-03 and 316L-04. With less overlapping molecular isotopes, the SIMS spectra of 316L-04 was examined first as there is more confidence in the assigned fragments.

When assigning the more complicated 316L-01 spectrum, fragments at the low and high mass ends of each cluster were examined. Peaks at the ends of the cluster are least likely to have overlapping molecular isotopes from different species. Therefore, one has increased confidence in their assignment as the likely hood of other molecular isotopes overlapping at the same mass is low. These peaks are listed in Table S1. $CuNiH_3^-$ is proposed to be present as the ratio of its molecular isotopes fit with the experimental data well. Either $CrCl_2^-$ or $CuNiH^-$ is present as these species have similar mass molecular isotopes which cannot be differentiated by the resolution of the ToF-SIMS machine. The ratio of the molecular isotopes for $CrCl_2^-$ has a better fit with the experimental data but the mass deviation of the $CuNiH^-$ molecular isotopes is lower than the corresponding $CrCl_2^-$ molecular isotopes. With the combination of $FePO_2^-$, $NiPO_2^-$, $CuNiH_3^-$, $NiCl_2^-$, $CuCl_2^-$ molecular isotopes and either $CrCl_2^-$ or $CuNiH^-$, the intensity of all significant peaks for the 115-140 u cluster are accounted for.

Table S1 Peaks assigned with confidence in 316L-01 ToF-SIMS spectrum

Nominal mass of peak u	Assignment
119	FeO_2
121	$NiPO_2$

Figure S6 Positive data set PCA scores plot for PC1 Vs. PC3. The explained variance for the total data set for each PC is given in brackets

132	$^{60}Ni^{37}ClCl$
133	$CuCl_2$
157	CuNiHCl

<u>PCA Peak List Selection</u>

The selection of the peak list for PCA analysis is an important step in ensuring all linear relationships are identified. A variety of approaches are taken when selecting the peak list for PCA analysis. Some use a hand-picked list from selected significant fragments whereas others use a threshold based on peak counts and signal to noise ratios (S/N-ratio) to select their peak list. Peak lists for positive and negative spectra were acquired using the peak search tool in Surface Lab 6. All peaks in the m/z range of 1-800 u that showed significant intensities (> 500 counts, S/N-ratio >2.0) were selected. In addition to this selection, any identified peaks which had low intensities and were not in the selection criteria, for example low abundance molecular isotopes, were also added. With this peak selection method, a large, representative sample of peak intensities was taken from the spectrum whilst ensuring all characteristic mass fragments are still included.

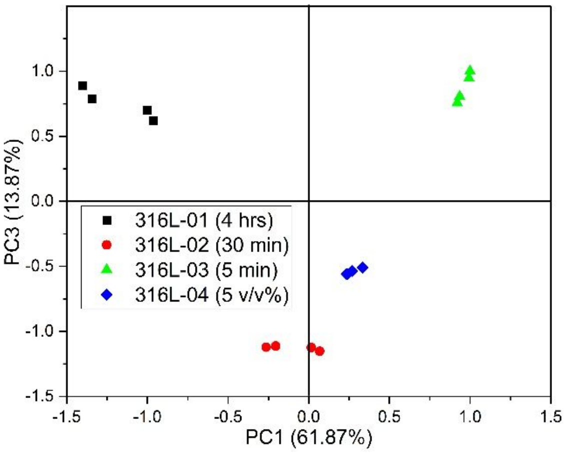

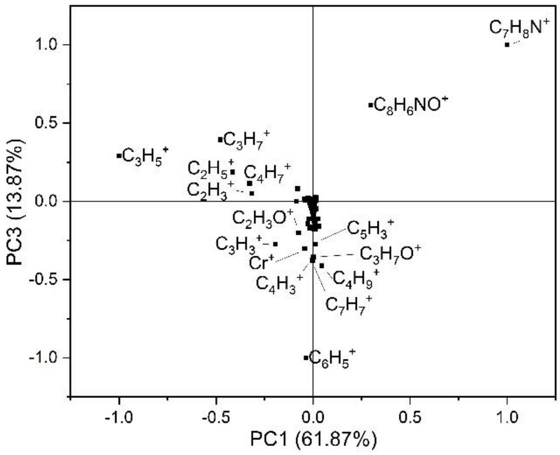

Figure S7 Positive data set PCA loadings for PC1 Vs. PC3. The explained variance for the total data set for each PC is given in brackets

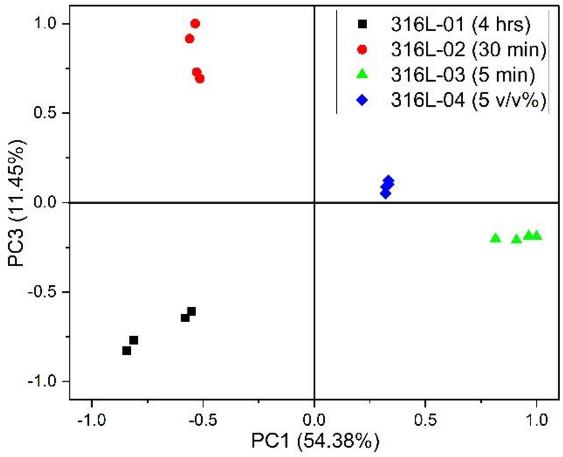

Figure S8 Negative data set PCA scores plot for PC1 Vs. PC3. The explained variance for the total data set for each PC is given in brackets

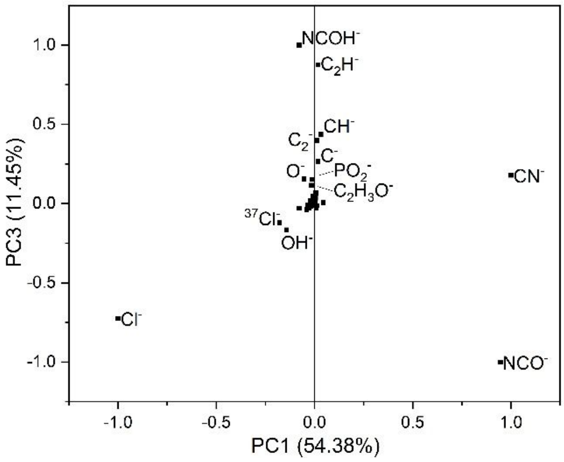

Figure S9 Negative data set PCA loadings for PC1 Vs. PC3. The explained variance for the total data set for each PC is given in brackets
